# Fjord oceanographic dynamics provide refuge for critically endangered *Pycnopodia helianthoides*

**DOI:** 10.1098/rspb.2024.2770

**Published:** 2025-04-02

**Authors:** Alyssa-Lois Madden Gehman, Ondine Pontier, Tyrel Froese, Derek VanMaanen, Tristan Blaine, Gillian Sadlier-Brown, Angeleen M. Olson, Zachary L. Monteith, Krystal Bachen, Carolyn Prentice, Margot Hessing-Lewis, Jennifer M. Jackson

**Affiliations:** ^1^ Hakai Institute, Calvert Island, British Columbia, Canada; ^2^ Institute for the Oceans and Fisheries, The University of British Columbia, Vancouver, British Columbia, Canada; ^3^ Central Coast Indigenous Resource Alliance, Campbell River, British Columbia, Canada; ^4^ Fisheries and Oceans Canada, Institute of Ocean Sciences, Sidney, British Columbia, Canada

**Keywords:** sea star wasting disease, infection, marine, refuge habitat

## Abstract

Disease outbreaks as a driver of wildlife mass mortality events have increased in magnitude and frequency since the 1940s. Remnant populations, composed of individuals that survived mass mortality events, could provide insight into disease dynamics and species recovery. The sea star wasting disease (SSWD) epidemic led to the rapid >90% decline of the sunflower star *Pycnopodia helianthoides*. We surveyed the biomass density of *P. helianthoides* on the central British Columbia coast before, during and after the arrival of SSWD by conducting expert diver surveys in shallow subtidal habitats from 2013 to 2023. We found a rapid decline in biomass density following the onset of SSWD in 2015. Despite consistent recruitment post-outbreak to sites associated with outer islands, we found repeated loss of large adult individuals over multiple years. Within nearby fjord habitats, we found remnant populations composed of large adult *P. helianthoides*. The interaction of temperature and salinity with the biomass density of *P. helianthoides* varied by location, with high biomass density associated with higher temperatures in the outer islands and with lower temperatures and higher salinity in the fjords. These patterns suggest that fjords provide refuge from consequences of SSWD and protecting these populations could be imperative for the species.

## 1. Introduction

Emerging infectious diseases can cause wildlife mass mortality events, and these events have increased in magnitude and frequency worldwide [1,2]. Mass mortality events can lead to profound ecological consequences, from rapidly altered species interactions to the large-scale loss of individual species [3,4]. Long-term baseline information is imperative to evaluate the extent of mortality events and the potential for species and community recovery [5]. Indeed, the variability in recovery across habitats, geographic locations or time could shed light on the drivers of the original event and the potential for recovery. Depending on abiotic or biotic factors that influence the transmission potential of a disease agent, certain areas could become a refuge from disease [6]. However, some areas could survive a disease outbreak purely by chance, necessitating causal or correlative links between abiotic and biotic conditions, remnant populations and disease mechanisms before applying the term ‘refuge’ to areas holding remnant populations. Conditions associated with remnant populations could therefore provide important information about the factors influencing disease dynamics.

Disease is the integrated outcome of interactions between pathogens or parasites, their hosts and the environment they inhabit [7]. A range of environmental conditions can influence the outcome and dynamics of host–parasite interactions, including temperature, rainfall, nutrients and salinity [6]. For example, many marine parasites can have reduced tolerance to low salinity compared with their hosts, creating a refuge from disease in low salinity conditions [8]. Variability in thermal tolerance can also create areas of refuge, as well as areas of increased risk of disease outbreak or spread [9,10]. These mismatches between the tolerance of either host or parasite to environmental conditions can create areas of refuge from disease [6,11]. Thus, especially for geographically broad disease-driven mass mortality events, there are likely to be areas of disease refuge. Taking into account any known environmental tolerances of the host or pathogen could inform likely areas of disease refuge. In marine systems, temperature and salinity stand out as likely drivers of disease refuge [8,11].

Sea star wasting disease (SSWD; also known as sea star wasting syndrome and asteroid idiopathic syndrome) caused a mass mortality event for a range of sea star species from Baja California to Alaska, starting in 2013 [5,12–16]. Although originally hypothesized to be driven by a potential virus [12], the causative agent remains elusive [12,17,18]. Several studies have suggested that microbial dysbiosis is associated with wasting signs [19–21], however, whether dysbiosis is a causative driver or a response following some other trigger remains unresolved. Indeed, another hypothesis suggests that the trigger for wasting signs could be low oxygen conditions that cause the proliferation of anaerobic bacterial growth in the diffusion boundary layer [20]. However, this hypothesis does little to explain how wasting could have devastated sea stars on exposed rocky shores [5], where high water movement remains, even at near-substrate levels within the boundary layer, which would maintain high oxygen concentrations [22].

Positive temperature anomalies are implicated as a potential trigger or exacerbation factor in SSWD [5,14,16], which suggests that areas with consistent or reduced temperature could be refuges from disease spread. However, not all outbreaks were associated with high temperatures [23] and it does not explain the loss of *Pycnopodia helianthoides* populations in deeper, cooler waters on the outer coast of California [14]. Disease modelling to date suggests that the best explanation could be the combination of disease spread and environmental triggers, namely temperature, that lead to localized outbreaks [16]. The association between SSWD and anomalous high temperature suggests that areas with comparatively cool water temperature within a region could provide a disease refuge.

Given that sea stars have little ability to osmoregulate they tend to avoid areas outside of their salinity tolerance (e.g. [24,25]). However, the limited information we have on the relationship between SSWD and salinity is notable. A few reports suggest that low salinity can exacerbate the disease in both *P. helianthoides* and *Leptasterias sp* [25,26]. Separate from SSWD, low salinity conditions are associated with decreased physiological responses in sea stars, and while there is some scope for acclimatization, there appear to be limits [24]. Thus, we expect that sea stars will avoid low salinity and thus variation in salinity could be associated with variation in SSWD outbreaks along the coast and disease refuge

Prior to the start of the SSWD outbreak, *P. helianthoides* was an abundant and dominant benthic predator in the intertidal and subtidal of the Northeast Pacific. Ubiquitous and charismatic, there was little indication before the SSWD outbreak that this species was at risk. Despite this, following the disease outbreak, *P. helianthoides* lost over 90% of its global population, has now been listed on the IUCN Red List as critically endangered [27], and is under consideration for being listed as threatened under the US Endangered Species Act [28]. The decline and persistence in loss of *P. helianthoides* varied across latitudes, with *P. helianthoides* functionally extirpated from its southern range (OR, CA and Mexico), and only remnant populations remaining in the northern range [27,29]. British Columbia (BC) had large documented losses during the original outbreak of SSWD, but pockets of remnant populations of *P. helianthoides* remain throughout the province [29].

**Figure 1.**
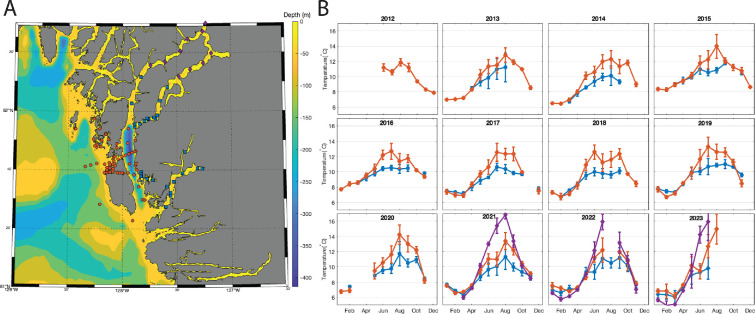
(A) Location of oceanographic sampling stations. Blue squares are stations that were located within fjords. Orange circles are stations that were located outside of fjords (outer islands). Purple triangles are stations where the salinity at 5 m was less than 20 salinity units in all years that were sampled. (B) Monthly average temperature at 5 m at sites around the central coast of BC from 2012 to 2023. Data were separated into fjord (blue squares), outer island (orange circles) and estuary (purple diamonds) stations. Error bars represent the standard deviation. Monthly averages were only calculated when at least three stations were sampled in a given month. Salinity patterns at these sites are in electronic supplementary material, figure S1.

The central BC coastline is a complex mix of mainland shore, fjords and distributed outer coast islands creating a large range of oceanographic properties and marine habitats. From its most southern to its most northern location, the BC coast spans across approximately 954 km, however, due to its complex geography, its total length is much longer, with approximately 4738 linear kilometres per degree of latitude [30]. With isolated islands and deep coastal incisions, as well as proximity to the mainland coastal mountains, BC’s climate varies drastically across the province. Several fjords are present on the central coast of BC with the longest measuring 170 km from the head of Dean Channel to the mouth of Fitz Hugh Sound. Fitz Hugh Sound separates Calvert Island, a large island exposed to the open ocean and part of the Hakai Lúxvbálís Conservancy, from the mainland (figure 1A). Most of these fjords have deep basins (up to 600 m) landward of a shallow sill (ranging from 40 to 300 m deep) and significant freshwater input at the head. During winter months, cold air and strong winds deepen the surface mixed layer [31], whereas over the warmer seasons, the influx of freshwater stays at the surface (approx. 5 m) and can create a current flowing out towards the open ocean [31]. The variance in salinity and temperature within the fjords contrasts with the relatively stable salinity and high surface temperatures found in the adjacent islands along the outer coast of BC.

We address the question of why we see variation in *P. helianthoides* population response to SSWD within the central coast of BC [29] by evaluating distribution, size and SSWD outbreaks from 2013 to 2023. We further evaluate the drivers of change in *P. helianthoides* biomass density from 2013 to 2023. We hypothesize that temperature and salinity drive variance in *P. helianthoides* population response, specifically we hypothesize that:

1. Sites and regions with high temperatures have increased the progression of SSWD and thus increased *P. helianthoides* population-level mortality following disease outbreaks.
2. Locations with low salinity have a lower density of *P. helianthoides*.
3. Salinity and temperature will interact to influence *P. helianthoides* populations, with higher mortality from SSWD in areas with high temperature and high salinity.

## 2. Methods

Within the central coast of BC, we evaluate *P. helianthoides* response to SSWD in the region and evaluate drivers of areas of apparent refuge from SSWD. To do this, we (i) evaluate the variability in oceanographic conditions found within the region, (ii) evaluate the effect of SSWD on *P. helianthoides* size distribution and biomass density over time, (iii) describe the timing and geographic location of SSWD outbreaks and (iv) test hypothesized drivers of variability in the remnant and/or recovering populations of *P. helianthoides* within the region.

Researchers at the Hakai Institute and collaborators have been conducting biodiversity surveys within nearshore habitats on the central coast of BC since 2013. Sea stars were quantified as a part of independent projects based on different focal species or habitats (e.g. kelp, seagrass, etc). While each project has specific methods, all involve overlapping personnel and repeated surveys of single locations over time. Complementarily, the Central Coast Indigenous Research Alliance (CCIRA) conducted snapshot surveys throughout the region and quantified sea star density using similar survey techniques and some overlapping personnel. The Wuikinuxv, Kitasoo/Xai’xais, Heiltsuk and Nuxalk First Nations hold indigenous rights to their territories, where all data were collected. Our work was done with formal agreements, review and collaboration with each nation.

Throughout the surveys, all sea stars showing signs of disease were reported. Sea star species have variable timelines in response to disease exposure in the lab [15], and *P. helianthoides* show signs of disease for a short window of time (1–4 days) before mortality and disintegration (unpublished data). With such a short time window to show and document signs of disease, we assume that any documented wasting in *P. helianthoides* is a small subset of the diseased stars within the region. Mortality following SSWD tends to be size dependent [32], with larger stars dying at higher rates than smaller stars. For the above reasons, we use changes in biomass density and size distributions to detect the effects of SSWD on *P. helianthoides*, in addition to prevalence scores.

### (a) Oceanographic data collection

Temperature, salinity and pressure (CTD) and oxygen sensor profiles have been collected at weekly to annual time scales since June 2012. Data were collected with either a Seabird 19 plus CTD or an RBR CTD and oxygen was measured by either a Seabird SBE43 or Rinko III oxygen sensor. CTD and oxygen sensor data were processed using Seabird’s SeaSoft software or following the RBR processing steps recommended by Halverson *et al*. [33]. For this research, oceanographic station data collected between June 2012 and August 2023 were analysed if they fitted the following criteria: (i) the water depth was greater than 5 m, (ii) the station was sampled at least five times and (iii) the station was sampled for at least two different years. In total, 4417 CTD and oxygen profiles fitted our criteria and were analysed. Temperature, salinity and oxygen at 5 m depth were examined to determine if there was a spatial difference between stations located within fjords (blue squares in figure 1) and outside of fjords (orange circles in figure 1). An initial examination found that there were some stations located near the head of Dean and Burke Channel where waters at 5 m were much fresher and warmer than in other regions. Thus, a third category, which we call estuary, was defined as one where the salinity at 5 m was fresher than 20 ppt at least one time in each year that was sampled (purple diamonds in figure 1).

The data sampling frequency was inconsistent at each station. To minimize any bias associated with sampling frequency, the monthly average 5 m temperature, salinity and oxygen was first calculated at each station (if data were collected more than once in any given month). Then, the monthly average for each station was used to calculate a monthly average for each region (i.e. fjord, outer island and estuary) for each year. The monthly average was only calculated for each region if at least three stations were sampled in that region during that month. The upper thermal threshold for *P. helianthoides* has been reported as 14℃ [34]. To compare regions, or location category, the percentage of stations in each month where temperatures exceeded 8℃, 10℃, 12℃ and 14℃ was calculated for both fjords and outer island regions (electronic supplementary material, figure S2).

### (b) Biological data collection

#### (i) Subtidal surveys

All subtidal surveys (associated with seagrass, kelp, rocky reef, electronic supplementary material, table S1), except herring spawn surveys, were conducted using similar methods. All sea stars within a 1 m swath along both sides of a 30 m long transect tape positioned along a targeted depth, parallel to shore, were identified to species and radius (longest arm) measured to the nearest 1 cm by scuba divers for a total surveyed area of 60 m^2^ per transect. Starting in 2015, a wasting score between 0 and 4 was assigned to each individual [5,35]. We replicated transect lines within the site between three and six times, by depth or along the same depth contour, but varying in location, depending on the project (electronic supplementary material, table S1).

While the transect methods remained the same between sites and projects, the method of replication over time varied by project. Sites associated with the Rocky Reef project had repeated transects at the site level, so transects were done within the same general area but varied in exact location. Transects associated with the Kelp and Seagrass projects had permanent transects that were replicated over time.

#### (ii) Unique surveys

##### Central Coast Indigenous Research Alliance rockfish survey

While the transect methods are the same as those from other subtidal transects, the methods for quantifying sea stars associated with the CCIRA rockfish surveys changed over time. In 2018 and 2019, *P. helianthoides* were anecdotally noted, and sizes were estimated. In 2020, *P*. *helianthoides* were added to the official survey methods, so for 2020−2023 all individuals were noted and measured to the nearest centimetre. To maximize geographical coverage, the sites associated with the CCIRA Rockfish project were not replicated, with each site visited a single time in a snapshot survey. In some cases, a general area was re-surveyed multiple years in a row.

##### Herring survey

We conducted subtidal surveys on rocky substrates at locations with herring spawns in the spring of 2014, 2015, 2016 and 2019 using transects perpendicular to the shore that varied in length based on the spawn width. Five quadrats were evenly placed along the length of the transect. All sea stars within the quadrats were identified to species and measured to the nearest centimetre. The depth and dominant substrate type for each of the quadrats was also recorded. In 2015, sea stars within 1 m swaths on either side of the perpendicular transect between each quadrat placement were also accounted for. We used data from this survey type to evaluate size distributions and observe wasting events, as the quadrat-based sampling design produced highly variable biomass densities compared with the belt transect design.

### (c) Statistical analysis

To compare data between different projects, we calculated *P. helianthoides* biomass density (kg per 10 m^2^) at the transect level. We calculated biomass using a length–biomass conversion [36]. We grouped the data based on their distinct oceanographic setting: shallow subtidal within a fjord and shallow subtidal associated with the outer islands. In most analyses below, we include location (i.e. fjord or outer island) as a predictor. All data were analysed using R v. 4.2.2 and RStudio v. 2023.06.0+421 [37,38]. Visualization used several R packages, including *ggplot*, *cowplot, gratia* and *visreg* [39–41].

#### (i) Size distribution

We evaluated size distributions across two time scales. First, to address whether SSWD mortality was variable by size, we evaluated the change in the upper limit to *P. helianthoides* size over the year that SSWD was first documented in the region. We used all surveys conducted in 2015, and fitted a quantile regression with size as the response variable, evaluating the upper limit of size over this time period with the 0.90 quantile [42,43]. Quantile regression was visualized using *visreg* [44]. Second, we explored the variation and response of size distributions over time and space. We included all survey types and years in this second analysis of size, which means that sites were sampled unevenly over time and space (electronic supplementary material, table S1). Thus, to evaluate whether geographic location or year altered size distributions we used non-metric multidimensional scaling [45]. Because we were using datasets that varied in their sampling design and sampling frequency, we lumped all sizes measured within-year across sites, habitats and survey type. To run the analysis, we transformed the size distribution data so that each year and unique location was a row, and sizes were columns, with a count of how many individuals were in each size grouping. Size groupings were to the nearest centimetre. We fitted a permutations analysis of variance test (vegan, *adonis*) to evaluate whether wasting time period (year) and location (fjords or outer islands) influenced the size distribution of individuals [46,47].

#### (ii) Outbreak timing

To evaluate the timing of sea star wasting disease in our datasets, we observed the frequency distribution of wasting scores over time. Signs of sea star wasting disease are remarkably short lived in *P. helianthoides*, with individuals going from the earliest signs of disease to mortality and disintegration within days (unpublished data). Thus even a monthly survey regime could miss the outbreak timing within a region. Other species of sea star, such as *Pisaster ochraceus* and *Evasterias troschelli*, show signs of disease over longer time periods [15]. As such, in order to mitigate against missing disease outbreaks, we combined observations of wasting from any species of sea star in the region to observe the patterns in wasting over time. We report levels of wasting for all stars combined. For visual observation, we included all survey types across the full datasets, which makes the data unsuitable for formal analysis.

#### (iii) Outer island biomass density across the timing of the outbreak

To evaluate the response of *P. helianthoides* from before, during and after the disease outbreak, we selected a subset of sites that were monitored roughly evenly over time (electronic supplementary material, table S2). We fitted a generalized additive model (GAM, mgcv, *gam* [48,49]) to evaluate change in *P. helianthoides* biomass density over space and time. We fitted a geographic interactive smooth with latitude and longitude to account for the spatial distribution of sites, a numeric date smooth to account for a continuous time through the experiment, a month smooth to evaluate seasonality and a transect ID smooth as a random effect to account for survey design. We included the depth bin (shallow < 5 m, mid ≥ 5 m < 10, deep ≤ 10 m) as a parametric value. We fitted the model with a tweedie distribution, and the smoothness parameters were estimated using restricted maximum likelihood [49]. We evaluated the model for auto-correlation, and modelled the residuals using an auto-regressive moving average if necessary [50]. We evaluated the fitted model for all model assumptions, including concurvity and auto and cross-covariance.

#### (iv) Drivers of biomass density post-wasting

To test the hypothesis that temperature and salinity are driving variability *P. helianthoides* response to SSWD, and thus *P. helianthoides* change in biomass density over time, we worked with a subset of sites that had paired CTD measurements associated with the surveys (electronic supplementary material, table S3). We used general additive mixed effects models (mgcv *gamm* [48,49]) to evaluate the effect of temperature, salinity, geographic location and date on *P. helianthoides* biomass density. We included location category (fjord or outer island) as a main effect, and a smooth for month to evaluate seasonality. We included an interactive smooth of latitude and longitude. To evaluate the interaction between a smoothed effect and a main effect, we included ‘by’ factors within the smooth. We included the interaction between date and location category, with a smooth for date and location category as a by factor, and an interactive smooth for temperature and salinity with location category as a by factor. The original model design included depth, but the relationship between depth, temperature and salinity precluded including all three variables in the model, so we selected to include temperature and salinity. The model fit and evaluation of auto-correlation were examined the same as the biomass density models. Figures were created using *gratia* [39].

## 3. Results

Across all surveys from 2013 to 2023, we counted 5794 *P*. *helianthoides* individuals. The minimum size *P. helianthoides* recorded had a radius of 0.5 cm, the maximum size was 75 cm and the mean size was 10.90 cm. SSWD was first documented anecdotally around Calvert Island in the winter of 2015 [51]. We found individuals in all wasting categories, and SSWD prevalence varied from 0 to 100%.

### (a) Oceanographic patterns

Monthly average temperature at 5 m showed clear differences between the regions, particularly in the summer months (figure 1; May through September). In fjords, the average monthly temperature range was higher in the waters associated with the outer islands than within the fjords (table 1). Estuary waters were primarily sampled from 2021 to 2023. During these years, the coldest monthly average (9.0 ± 0.2℃) was observed in May 2022 while the warmest monthly average (16.8 ± 0.6℃) was observed in August 2021.

**Table 1.** Monthly average water temperature at 5 m at sites with the fjords and adjacent to outer islands within the central BC coast.

month	fjord	outer islands
May	8.6–10.0℃	8.7–10.9℃
June	9.3–11.0℃	9.4–12.6℃
July	9.7–11.1℃	10.5–13.3℃
August	9.6–11.7℃	11.4–15.0℃
September	9.3–11.7℃	11.2–13.0℃

While the percentage of stations that observed at least 8℃ was similar between regions, waters around outer islands regularly exceeded 10℃, sometimes exceeded 12℃ and rarely exceeded 14℃ (electronic supplementary material, figure S2). In general, the warmest average years around outer islands were 2015, 2019, 2020 and 2023. In fjords, temperatures often exceeded 10℃, rarely exceeded 12℃ and only once exceeded 14℃ (electronic supplementary material, figure S2). In general, the warmest years in fjords were 2015, 2016, 2019 and 2020.

### (b) Size distribution

**Figure 2.**
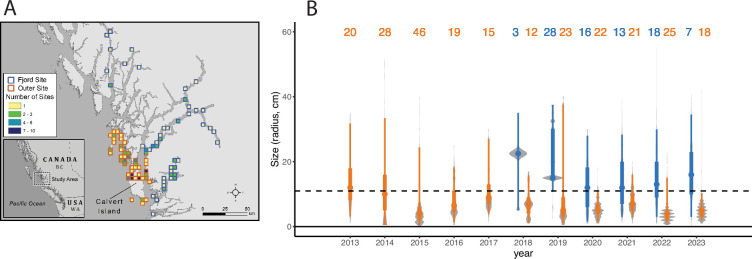
(A) Biological survey locations and (B) size distribution over time at all sites surveyed within fjords (blue) and outer islands (orange) around the central coast of British Columbia. Points indicate the mean population size, thicker lines indicate the interquartile range, the thinner lines indicate the 95% quantile and the grey area indicates the density of data around that point. The dashed line indicates the estimated minimum size at reproduction for *P. helianthoides* (S. Gravem & J. Hodin 2025, personal communication). Numbers along the top indicate the number of sites surveyed each year, with colours indicating the location category.

The upper limit of *P. helianthoides* size declined across the season in 2015 (quantile regression, τ = 0.90, coefficient = −0.10, *p* < 0.001; electronic supplementary material S3). It should be noted that quantile regression is sensitive to sample size, and the number of *P. helianthoides* encountered declined over this time period, so the test could overestimate the level of loss. The maximum *P. helianthoides* size declined from 40 cm radius in April 2015 to 4 cm in September 2015 (electronic supplementary material, figure S3). There was no change in the lower limit of *P. helianthoides* over the same time period (quantile regression, value = 0.0 , *p* = 1.0). Comparing across all years, we found that the size distribution of *P. helianthoides* varied by year (figure 2; permanova, sum of squares = 0.9272, *R*^2^ = 0.04781, *F* = 3.4471, *p* = 0.001), by location category (figure 2; electronic supplementary material, figure S4, fjord or outer island; permanova, sum of squares = 1.2387, *R*^2^ = 0.06387, *F* = 4.6053, *p* = 0.001), and by the interaction between location category and year (permanova, sum of squares = 1.0898, *R*^2^ = 0.05619, *F* = 4.0516 *p* = 0.001). The residual variance was large (permanova, sum of squares = 16.1386, *R*^2^ = 0.83213). At sites associated with outer islands, the mean individual size was lower than the reported minimum reproductive size of *P. helianthoides* (S. Gravem & Hodin 2025, personal communication) from 2015 to 2023 (figure 2B). Within the fjords, the mean individual size was greater than the minimum reproductive size for all years documented, from 2018 to 2023 (figure 2B). The number of sites visited varied by year and location (figure 2B), with the lowest number of sites from the first year of observed data in the fjords in 2018.

### (c) Outbreak timing

**Figure 3.**
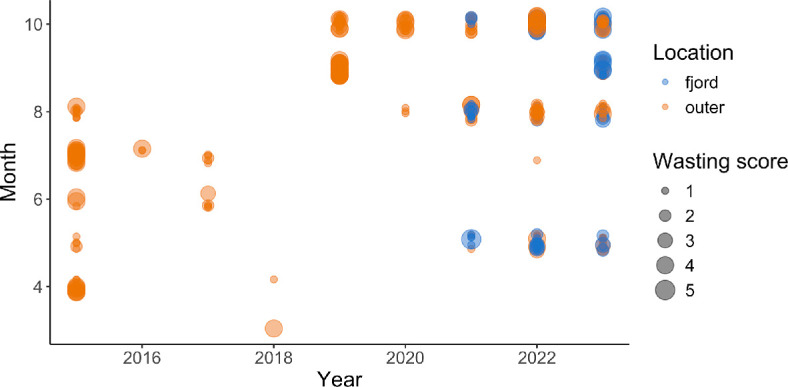
Occurrence of wasting sea stars, by SSWD score, estimated by divers during subtidal sea star surveys by month, for all species of sea star showing signs of disease. Wasting scores are categorical from 1−4 [5,35]. Colours indicate whether the observations were associated with sites in the outer island or within the fjords. Only data with positive disease detection are shown, so gaps in months represent time periods without surveys or disease detection, rather than an absence.

We first observed SSWD in 2015 and consistently found evidence of wasting in all years following (figure 3). We found wasting stars in sites associated with the fjords and associated with the outer islands (figure 3). In the year of the first outbreak, wasting stars were documented through all months with surveys, from April to August. In the later years, while wasting was documented in most months, there was an increase in observations of wasting individuals in August and October (figure 3). We documented signs consistent with wasting in 15 species, including *P. helianthoides*, *Henricia sp*., *Mediaster aequalis*, *Evasterias troschelii*, *Dermasterias imbricata*, *Orthasterias koehleri*, *Crossaster papposus*, *Stylasterias forreri*, *Pisaster ochraceus*, *Luidia foliolata*, *Patiria miniata*, *Solaster stimpsoni*, *Pisaster brevispinus*, *Hippasteria spinosa* and *Leptasterias sp*.

### (d) Outer island biomass density across the timing of outbreak

**Figure 4.**
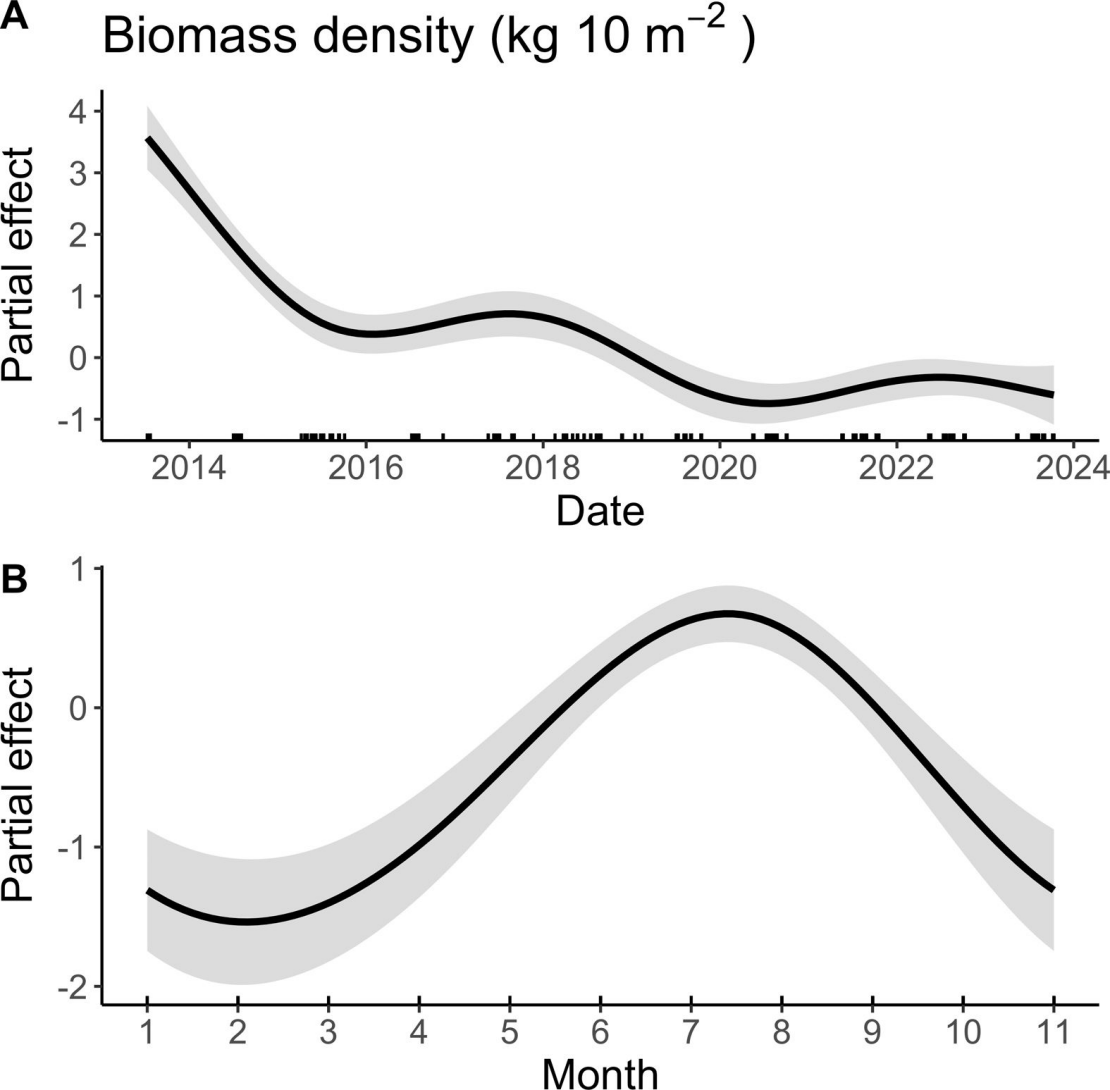
The partial effect of time (A) and month of year (B) on *Pycnopodia helianthoides* biomass density (kg per 10 m^2^) from 2013 to 2023. The rug marks on the *x*-axis in (A) indicate survey dates.

We found evidence of auto-correlation in our GAM, so we modelled the residuals using an auto-regressive moving average with an AR (*p* = 0, *q* = 3) correlation structure, which was the correlation structure with the lowest AIC value. The GAM fit had an AIC = 9448.61, *R*^2^ = 0.46 and a scale estimate = 0.66. The geographic location (latitude and longitude), transect ID, date and month all significantly affected biomass density (table 2; figure 4). In addition, there was a significant difference between shallow and deep depth bins (shallow > 5 m, deep = 10–16 m, table 2). We found a marked decline in *P. helianthoides* biomass density in 2015, from 2015 to 2018 there appeared to be a slight recovery, followed by a second decline around 2019 (figure 4; table 2). We found that biomass density was highest in the summer, peaking around July and August (figure 4; table 2).

**Table 2.** Generalized additive model evaluating the effect of binned depth (shallow < 5 m, mid = 5–10 m and deep=10–16 m), date, month, location and transect ID on the biomass density of *P. helianthoides*. The adjusted *R*^2^ = 0.457, the scale estimate = 0.66243 and *n* = 1887.

A. parametric coefficients	estimate	s.e*.*	*t-*value	*p*‐value
(intercept)	−5.377	0.1971	−27.2759	< 0.0001
depth bin (mid)	−0.3267	0.2152	−1.5178	0.1292
depth bin (shallow)	0.8719	0.2407	3.6229	0.0003

B. smooth terms	e.d.f.	ref.d.f.	*F*-value	*p*‐value
s(date)	4.8682	4.8682	40.3731	< 0.0001
s(month)	1.948	2	34.2633	< 0.0001
s(transect ID)	100.3352	400	0.5353	< 0.0001
s(latitude, longitude)	12.8928	12.8928	6.7936	< 0.0001

### (e) Drivers of biomass density post-wasting

**Figure 5.**
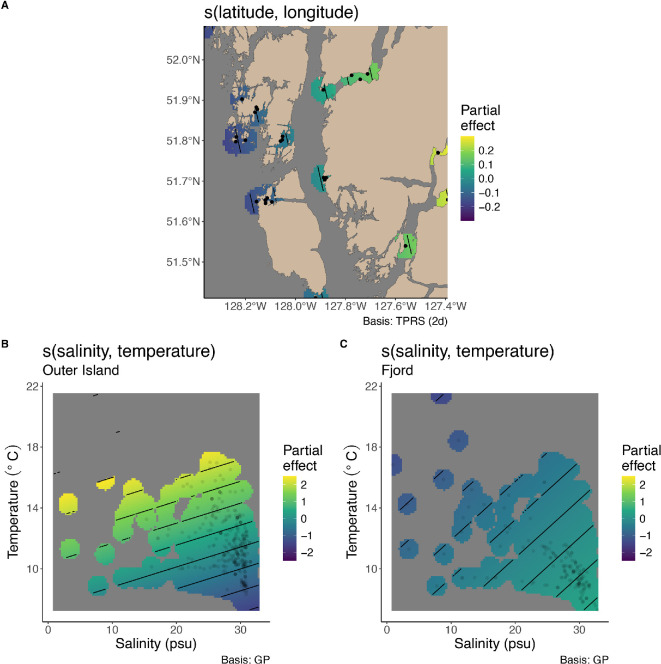
(A) The partial effect of geographic location on *P. helianthoides* biomass density (biomass density, kg per 10 m^2^) from 2021 to 2023, with points indicating location of surveys. (B) The partial effect of the interaction between temperature and salinity on *P. helianthoides* biomass density at sites within the fjords, with points indicating the conditions present at survey locations and (C) the partial effect of the interaction between temperature and salinity on *P. helianthoides* biomass density at sites in the outer islands. Partial effects from a GAM fit (table 3), with the smooth term and interaction and location indicated above the plot.

The GAM fit had an adjusted *R*^2^ of 0.34 and a scale estimate of 1.17. The geographic location had a significant effect on *P. helianthoides* biomass density, with the highest biomass density associated with the fjords (figure 5A; table 3). The interaction between salinity and temperature had a significant effect on biomass density in the outer island sites, with different relationships by location category. In the fjords, a non-significant trend towards the highest biomass density was found in water with high salinity and low temperature (figure 5C; table 3). In the outer islands, the highest biomass density was found associated with high salinity and high temperatures (figure 5B; table 3). Date had an effect on biomass density in the fjords, where biomass density increased from 2021 to 2023, but not in the outer islands (table 3).

**Table 3.** Generalized additive model results of (A) parametric coefficients and (B) smooth terms evaluating the effect of date, month, location category (location), the interaction between salinity and temperature by location category and geographic location on biomass density of remnant populations of *P. helianthoides*. The adjusted *R*^2^ = 0.34, the scale estimate = 1.17 and *n* = 472.

A. parametric coefficients	estimate	s.e.	*t*-value	*p*‐value
(intercept)	−1.85	0.36	−5.2	<0.01
location: outer island versus fjord	−3.44	0.55	−6.2	<0.01

B. smooth terms	e.d.f.	ref. d.f.	*F-*value	*p*‐value
s(date): location, fjord	1	1	4.22	0.04
s(date): location, outer island	1	1	1.75	0.19
s(month)	0.81	2	1.22	0.07
s(salinity, temperature): location, fjord	2	2	2.49	0.08
s(salinity, temperature): location, outer island	2	2	5.31	0.01
s(longitude, latitude)	2	2	0.15	0.86

## 4. Discussion

In the outer islands of the central coast of BC, a large decline in biomass density of *P. helianthoides* was observed the year SSWD was first detected. Recovery was apparent following the initial biomass loss, followed by a subsequent second decline. As found with other studies, there was a strong size dependency in mortality, especially in the year following the outbreak, with a pronounced loss of large individuals (e.g. maximum radius dropped from 40 cm to 4 cm from April to September). In the outer islands, despite multiple years of documented recruitment at some sites, the mean size remains below the minimum size for reproduction in 2023. In contrast to these dramatic shifts in outer coast island habitat, contemporary shallow subtidal habitats inside fjords house robust and stable *P. helianthoides* populations, with the full size distribution similar to that found in the outer island habitats pre-wasting. We observed signs of SSWD in fjords, but these observations of disease were not followed by a detectable decrease in overall biomass density. The interaction between salinity and temperature was predictive of *P. helianthoides* biomass density both within the fjords and the outer island habitats, however, the relationship differed between the two. The biomass density of *P. helianthoides* in both locations was associated with high salinity (> 15 psu), but in the fjords, biomass density was highest in cool water temperatures, and in the outer islands it was highest in the warmer water temperatures. Thus, the environmental conditions within the fjords appear to be providing a form of refuge from the consequences of SSWD.

**Figure 6.**
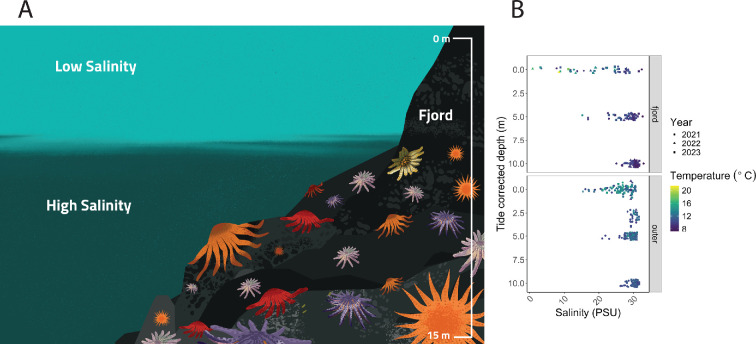
(A) Artistic illustration of the influence of the freshet on *P. helianthoides* distribution across depths. (B) Salinity and temperature across depth for data included in figure 5, illustrating that salinity on the outer islands is all above 15 psu, and that higher temperature water in the fjords is associated with fresher water.

*P. helianthoides* in fjord habitats appear to be responding differently to SSWD than those in other habitats and regions [29]. The contrast between the interaction between salinity and temperature on biomass density within the fjords and outer islands suggests that these habitats could be a refuge from disease. However, observed SSWD within the fjords indicates that a lack of SSWD exposure is unlikely to be the mechanism fostering this refuge. Instead, we suggest that the unique oceanographic conditions within the fjords, specifically through the increase in freshwater input during snowmelt, known as the freshet, could be keeping *P. helianthoides* in conditions that optimize host health and/or limit disease progression and transmission (figure 6A). Our results lead to the hypothesis that the freshet timing, alongside the summer months, could be reducing *P. helianthoides* exposure to hot summer water temperatures (figures 1, 5B,C, 6B and table 1). The sustained large influx of fresh water over the summer months creates an upper layer of low salinity, at times down to approximately 0 psu, that can be between 0 and 5 m in depth [52]. Although the salinity tolerance of *P. helianthoides* is unknown, they are associated with higher salinities [53], and related species have low salinity tolerances around 15 psu [24]. As such, low salinity could drive *P. helianthoides* deeper to avoid the upper few metres of water during the freshet, and thus remain separated from the high temperatures found in the shallow subtidal in late summer. Indeed, in the outer island where salinity > 15 psu is available throughout the water column (figure 6B), we observed the highest biomass density of *P. helianthoides* in warmer waters.

Temperature can have a nonlinear relationship with disease risk, such that the influence of changing temperatures on disease dynamics can be context and timing dependent [9,10,54]. For SSWD, cooler temperatures decrease the rate of disease signs and time to mortality for *P. ochraceus* [55]. Based on our observations of *P. helianthoides* within the fjord habitats associated with cooler conditions, we find support for the role of temperature in altering response to SSWD. However, the presence of disease at these lower temperature environments demonstrates that low temperature does not prevent disease transmission completely. Instead, these results suggest that lower temperatures could be altering one or several components of disease dynamics. We hypothesize that the SSWD *R*_0_, a metric that can model pathogen invasion potential in a susceptible population by integrating across host–parasite parameters, such as pathogen survival in the water and host susceptibility, is temperature dependent (e.g. [9,10]). If *R*_0_ is lower at cooler temperatures, that could explain the low incidence of disease and subsequent lower population mortality found within populations in isolated cool conditions within the fjords. Future modelling and host–pathogen physiological work is needed to evaluate this hypothesis. Importantly, the influence of temperature on disease dynamics likely varies with the timing and stage of the disease. Indeed, during the first SSWD outbreak in Oregon, the initial outbreak was associated with abnormally high temperatures, but prevalence continued to increase during subsequent cooler temperatures [23]. This could be explained by the phenomena observed in other disease systems, where the influence of temperature dependence decreases when large numbers of susceptible hosts are present [56].

Consistently high levels of recruitment post-wasting in the outer islands is encouraging and highlights questions about where these recruits are coming from. The consistency of sites with recruiting individuals and the visual connectivity between high recruitment sites and the fjords (figure 5A), suggests that the fjord remnant populations could be the main source of recruitment. Future work evaluating the oceanographic connectivity between these sites and the genetic relationships between locations is needed to explore this question further. However, despite consistent recruitment, the mean size of individuals has yet to recover eight years following the outbreak. In the first few years following wasting, there appeared to be growing cohorts, followed by a second loss of the extant larger individuals between 2017 and 2018 (figure 4). From 2019 to 2021 there was again some evidence of growing cohorts, which was again followed by apparent loss of extant larger individuals between 2021 and 2022 (figure 4). Wasting stars were documented in the years that we noticed the loss of biomass density (e.g. late summer 2017 and 2021). In fact, in late August/early September 2021 we documented the highest prevalence of wasting found in *Pisaster ochraceus* in the intertidal since 2015 (unpublished data). We hypothesize that these losses of cohorts indicate undocumented wasting events in these juvenile *P. helianthoides* communities. In both cases, the decline in body size distribution was after the third summer of growth for the juveniles, when the largest individuals of *P. helianthoides* within the cohort were reaching the size of reproductive maturity. Interestingly, in a cross-species evaluation of traits associated with costs from SSWD, the reproductive season was one of the few things that stood out as influential [57]. It is worth future research to explore whether reaching reproductive age increases the susceptibility of *P. helianthoides* to or reduces survival following exposure to SSWD.

There were some limitations to our datasets that should be addressed. Namely, we did not have pre-wasting surveys from within the fjord habitats. While there are relatively high biomass densities within the fjords compared with contemporary populations elsewhere [29], all of the data we have to evaluate biomass density within the fjords were collected post-wasting. Thus, it is possible that the biomass density found within the fjords could be a reduction or an increase from what was found in these habitats prior to the outbreak of SSWD. Furthermore, it is possible that the reduction in consequences of SSWD on *P. helianthoides* in the fjords could be due to individual or population resistance to disease, or altered microbial communities that defend against disease. Future work should evaluate these hypotheses.

If contemporary fjord conditions are creating a refuge for *P. helianthoides*, then the risk in the region to *P. helianthoides* populations remains high, as climate change effects on the freshet are altering the timing and extent in ways that could have dire consequences for these refuge populations. First, the area within the fjord with acceptable salinity levels is limited, with conditions becoming more estuarine near the head of the fjord (figure 1A). Second, winter Arctic outflow winds create a unique cool, salty, high oxygen subsurface water mass in some fjords, and as such are dependent on how climate change will influence the frequency of Arctic outflow events [58,59]. Third, Arctic outflow events may only influence fjords with an unobstructed connection with the mainland [59,60], so it will be important to evaluate which and how many fjord systems provide similar refuge conditions to *P. helianthoides*. To note, remnant populations of *P. helianthoides* are also found in Knight Inlet [61], which has a direct connection with the mainland [60]. Finally, the freshet, particularly in late summer, is driven primarily by glacial melt, a resource that is diminishing with climate change [62]. In sum, despite currently appearing to be a refuge for *P. helianthoides*, climate change and SSWD remain a threat to the survival of this species.

Our work documents a range of responses to a disease outbreak within a single species within a reasonably small geographic area in central BC. Remnant populations found within the fjord habitats and the association between abiotic conditions and host biomass density suggest that these fjords could be providing refuge from SSWD. Interestingly, other fjords within BC are home to remnant populations of *P. helianthoides*, suggesting that this could be a wider phenomenon [61]. If the drivers hypothesized in this study represent the field correctly, then protecting these populations within the fjords could be imperative for the recovery of this species. Evaluating the drivers of geographically variable mortality following SSWD enabled us to highlight important regions for species conservation and drivers of that variability. With wildlife mass mortality events on the rise [1,2], long-term monitoring of diverse communities with concurrent abiotic monitoring can help provide the data needed to outline potential mitigation and conservation actions.

## Data Availability

Data and code are available at [63]. Supplementary material is available online [64].
